# Extraction of Premature Newborns’ Spontaneous Cries in the Real Context of Neonatal Intensive Care Units †

**DOI:** 10.3390/s22051823

**Published:** 2022-02-25

**Authors:** Sandie Cabon, Bertille Met-Montot, Fabienne Porée, Olivier Rosec, Antoine Simon, Guy Carrault

**Affiliations:** 1 Univ Rennes, INSERM, LTSI-UMR 1099, F-35000 Rennes, France; sandie.cabon@univ-rennes1.fr (S.C.); bertille.met-montot@univ-rennes1.fr (B.M.-M.); antoine.simon@univ-rennes1.fr (A.S.); guy.carrault@univ-rennes1.fr (G.C.); 2 Voxygen, F-22560 Pleumeur-Bodou, France; olivier.rosec@voxygen.fr

**Keywords:** audio processing, spontaneous cry extraction, harmonic plus noise analysis, classification, real context, NICU, continuous monitoring, preterms, neuro-behavioral development

## Abstract

Cry analysis is an important tool to evaluate the development of preterm infants. However, the context of Neonatal Intensive Care Units is challenging, since a wide variety of sounds can occur (e.g., alarms and adult voices). In this paper, a method to extract cries is proposed. It is based on an initial segmentation between silence and sound events, followed by feature extraction on the resulting audio segments and a cry and non-cry classification. A database of 198 cry events coming from 21 newborns and 439 non-cry events was created. Then, a set of features—including Mel-Frequency Cepstral Coefficients—issued from principal component analysis, was computed to describe each audio segment. For the first time in cry analysis, noise was handled using harmonic plus noise analysis. Several machine learning models have been compared. The K-Nearest Neighbours approach showed the best results with a precision of 92.9%. To test the approach in a monitoring application, 412 h of recordings were automatically processed. The cries automatically selected were replayed and a precision of 92.2% was obtained. The impact of errors on the fundamental frequency characterisation was also studied. Results show that despite a difficult context, automatic cry extraction for non-invasive monitoring of vocal development of preterm infants is achievable.

## 1. Introduction

The main cause of mortality, pathology contraction and developmental disorders in neonates is being born prematurely [1]. In the seventies, dedicated care units called Neonatal Intensive Care Units (NICUs) were designed to provide a specialised medical care and ensure the optimal development of sick term and preterm newborns. Within these units, continuous monitoring methods based on electrophysiological signals have been integrated. They issue alarms and allow clinicians to react quickly in cases of vital function failures (apnea-bradicardia, oxygen desaturation, etc.). In order to go further in care, it is now time to propose similar methods to characterise the neurobehavioural development of newborns. Indeed, the current methods for monitoring newborn development are currently carried out on an occasional basis (e.g., sleep observations and ambulatory electroencephalography) and are thus not generalised. The Digi-NewB project, funded by the European Union programme for Research and Innovation Horizon2020, aims to meet this clinical need by offering new continuous monitoring solutions to help clinicians in their decision making [2]. To this end, this project proposes to carry out a multimodal analysis allowing the health of a newborn to be assessed by several criteria (vocal, motor, cardiorespiratory, etc.). In order to preserve the health and comfort of newborns, non-invasive modalities are considered.

In that context, the aim of the work presented in this paper is to propose audio analysis techniques to monitor the vocal development of preterm newborns in real NICUs. In particular, the focus is on automatic cry extraction, which is a step of the utmost importance to provide vocal development markers (e.g., frequency features).

Indeed, analyses of spontaneous cries have been shown to be an informative tool to assess the development of premature newborns (see [3] for a review). More precisely, the relationship between frequency content and increasing gestational age (GA) has been found important [4,5,6]. In particular, fundamental frequency ($F_{0}$) is generally higher in preterm infants than in full-term newborns at term-equivalent ages [5,6]. However, in these studies, the authors focused their analyses either after manual extraction of cry events [5] or on recordings made in controlled environments [4,6].

Extracting newborn cries is challenging due to the fact that in NICUs, sounds from various sources (e.g., alarms and adult voices) can occur [7]. Besides, conditions of recording differ regarding the GA and postmenstrual age (PMA) of each newborn (e.g., type of room and type of bed). To date, only a few studies tackled this problem, by means of a Hidden Markov Model (HMM) [8], Gaussian Mixture Model (GMM) [9] or Convolutional Neural Network (CNN) [10] using Mel-Frequency Cepstral Coefficients (MFFCs) as input features. Although the reported accuracies were high (91.1% [9], 89.2% [8], and 86.6% [10]), strong limitations regarding the representativeness of the training and evaluation datasets prevent them from being considered robust enough for deployment in clinics. Indeed, short audio recordings, limited recording environments and limited PMA and GA ranges of newborns were considered. An interesting proposition to counteract the lack of accessible real-world data for training was made in [10], where the generation of simulated data was proposed for training the CNN model. However, the scope of the proposed method is limited, since only one room was simulated, and the final model was only tested on a few sequences of 30 s of real-world data of a unique newborn. Thus, a method taking into account all the challenges of NICUs remains to be proposed. Such a solution will provide a robust continuous monitoring tool to improve the health care of newborns through cry analysis.

This paper is an extension of our previous conference paper in which we presented a new strategy to automatically extract spontaneous crying by newborns from long duration recordings taken in real NICUs [11]. The proposed method leans on segmentation of all sound events, feature engineering and cry classification. The materials and methods used to set up and evaluate the approach are presented first. Then, results are presented: the identification of the best classification model using an annotated database and the deployment of the full approach on long audio recordings. These are followed by a Discussion and a Conclusion sections. The main additions of this publication to previous work are as follows:

- The study of the contribution of each original feature to the principal components used for classification;
- A comparison between two training strategies for classification;
- An in-depth analysis of the impact of classification on fundamental frequency estimations for cry characterisation.

## 2. Materials and Methods

**Figure 1:**
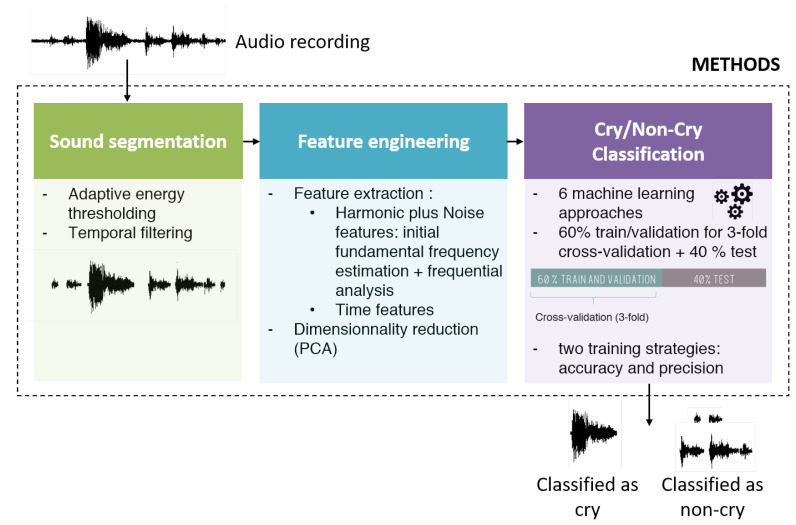
Overview of the cry extraction process.

Figure 1 illustrates the process of cry extraction based on machine learning techniques that is proposed in this paper.

In this section, databases that have been built to train and evaluate our approach are firstly presented. Then, the set of features used for the classification are described. Finally, the investigated classification approaches and the associated training strategies are introduced.

Experiments presented in this study were performed using Python 3.7.3. Machine learning developments were made with the support of scikit-learn 0.23.2.

### 2.1. Databases

Two databases were extracted from the Digi-NewB database: one to select the best classification model and the other to evaluate the performance of the selected model once deployed. Acquisitions took place in six French NICUs from 2016 to 2020 in a wide variety of bedding environments, i.e., incubators, tables and cradles. This study received ethical approval from the Ouest IV Ethics Committee (reference number 34/16), and at least one parent of each newborn gave signed consent for inclusion in the study. Audio streams were recorded with omnidirectional microphones (FG- 23329-P07 marketed by Knowles Acoustics) and stored in mono .wav files at 24 kHz (see [12] for more details about data acquisition).

#### 2.1.1. Annotated Database

A set of 27 30 min recordings, each representing a range of conditions encountered in NICUs, was selected. They involved fourteen boys and seven girls born between 27 + 5 and 41 + 4 GA and recorded between 28 + 5 and 41 + 5 PMA. Some of them were recorded two times in different acquisition environments.

From each audio recording, sound segments were firstly extracted using the sound segmentation step (see Section 2.2). We chose to annotate segments containing only one type of event, meaning that segments with overlapping sounds (e.g., cry with adult voice) were not selected. Then, based on previous works [7,13], 637 segments were annotated into 6 classes.

Sounds emitted by the newborns were divided into three categories: cries; vocalisations (e.g., cooing); and other baby noises, such as coughing or hiccups. The auditory differences between vocalisations and cries are subjective, and thus only obvious cries were annotated as such. Hence, 198 cries were extracted from all recordings except one of them, since no cry was found in it. The number of cries per recording ranged from 2 to 12 in the recordings containing cries. For the other two categories, baby vocalisations and baby noises, 93 and 65 segments, from all recordings, were, respectively, annotated.

Others sounds were also classified into three categories: adults voices (87 segments), alarms from devices (85 segments) and background noises (109 segments). The most diverse segments were selected to be part of each category. Thus, male and female voices; several types of alarms; and many background noises coming from the activity of adults (e.g., doors opening/closing, packaging friction or water flowing from a tap) and from devices (e.g., ventilatory support airflow and bed adjustment noises) were selected.

#### 2.1.2. Deployment Database

To deploy and evaluate the finally selected model, 42 recordings of 23 newborns (10 girls and 13 boys) of several GA (from 25 + 6 to 40 + 3) and PMA (from 28 + 1 to 41 + 3) were selected. The median duration of recordings was about 8 h, giving a total of 412 h.

### 2.2. Sound Segmentation

The first step of our approach is based on the unsupervised segmentation method proposed in [14]. This method leans on energy thresholding: two thresholds are successively estimated by the mean of the Otsu method to distinguish silences from noise-producing activities [15]. Through this step, all sound events are segmented. Thus, when applied to long audio recordings performed in NICUs, irrelevant sounds segments are also extracted, such as adult voices or alarms. To limit the number of extracted sounds events, only segments lasting between 250 milliseconds and 5 s were considered. This interval was chosen because of the experience we gained during our study, since no consensus on the minimal length of a cry event was revealed by the literature review [3]. In fact, in studies dealing with premature newborns, values ranged from 150 to 260 milliseconds [6,14,16].

### 2.3. Feature Engineering

The most relevant features for cry analysis were identified from a review of the literature on audio analysis in preterm newborns [3]. The underlying hypothesis is that an analysis focusing on features relevant to cry analysis will give discriminating values to classify cry and non-cry sounds. In the literature, time features, fundamental frequency and Mel-Frequency Cepstral Coefficients were mostly investigated [8,9,10]. Preprocessing steps were applied in order to reduce the effects of noise on $F_{0}$ and MFCCs estimations, such as beamformer [10] and signal decomposition [9].

In this study, we propose to handle noise by modelling audio segments using the harmonic plus noise model (HNM) that is usually applied in speech synthesis [17]. HNM analysis is suitable for quasi-harmonic signals, which is the case for baby cries and vocalisations, adult voices and alarms. From there, frequency features are estimated. They are supplemented by time features directly computed from the original signals. To finish, in order to limit the number of features for classification, a dimension reduction by means of principal components analysis (PCA) is performed. All these elements are detailed in the following sections.

#### 2.3.1. Harmonic plus Noise Model Features

The principle of HNM analysis is to create a synthetic signal s(t) composed of harmonic h(t) and noise n(t) parts that fit the original signal:

(1) s(t)=h(t)+n(t)

Indeed, the spectrum of voiced speech signal can be divided into two bands bounded by a maximum voiced frequency, a time varying parameter. The lower band is related to the harmonic part and the upper band to the noise part. The harmonic part h(t) is modelled as a sum of harmonics:

(2) $$h\left(t\right)=\sum_{k=1}^{K(t)}A_{k}\left(t\right)\cos\left(k\theta \left(t\right)+\Phi_{k}\left(t\right)\right)$$

where $A_{k}\left(t\right)$ and $\Phi_{k}\left(t\right)$ are, respectively, amplitude and phase at time *t* of the *k*-th harmonic. K(t) represents the time-varying number of harmonics included in the harmonic part. On its part, the noise part n(t) is supposed to have been obtained by filtering a white Gaussian noise u(t) by a time-varying, normalised all-pole filter f(t,τ) and multiplying the result by an energy triangular-like envelope function e(t), as follows:

(3) n(t)=e(t)[f(t,τ)∗u(t)]

To perform the analysis, it is first necessary to limit the analysis to a certain frequency band that we chose to adapt to cry analysis ($F_{min}$ = 150, $F_{max}$ = 750 Hz).

Secondly, to fit the model, an initial estimation of $F_{0}$ must be provided. In this study, it is estimated by the mean of the continuous wavelet transform, as proposed in [18], designed for the analysis of newborn crying. Once the signal is modelled, the spectral conversion of h(t) is performed to extract MFCCs. The analysis is performed over an audio segment on frames of 5 milliseconds without overlap. The audio segment is then summarised by median values of each feature coming from HNM analyses of each frame.

#### 2.3.2. Time Features

Two time features have been also defined to characterise the segments:

- Duration, in seconds, of the segment since some events may last longer (e.g., adult speech) or take less time (e.g, beep) than typical cries;
- Zero Crossing Rate (ZCR), already shown to be useful to distinguish alarms from cries [19,20]:
  (4) $$ZCR=\frac{1}{T-1}\sum_{i=1}^{T-1}1_{R<0}\left(s_{t}s_{t-1}\right)$$
  where *T* is the length of the signal s(t) and $1_{R<0}$ is the indicator function.

#### 2.3.3. Synthetic Resume of the Set of Features

In total, 124 features were computed. In our case, some of these features kept null values for all the audio segments studied (e.g., related to high harmonics and issued from HNM analysis) and have been discarded from the final feature set. Table 1 synthesises the remaining 73 features.

The physical meaning of each of these features sometimes strongly depends on the source of the sound (e.g., speech, device). For example, in the case of voiced sounds (i.e., produced by newborns and adults), the fundamental frequency corresponds to the speed of opening and closing of the glottis in the vocal tract, whereas it is a configuration of a device when it is a question of alarm. For their part, the number of harmonics, the harmonic amplitudes and the harmonic phases represent the harmonic complexity of the sound and characterise the capacity of the newborn or the adult to produce a complex sound, but also the complexity of a configured alarm. The gain and filter coefficients are used to model the noisy parts of all types of sound regardless of their sources, but are supposed to be more discriminative for background noises. However, some features have more generic meanings. Indeed, the Mel-Frequency Cepstral Coefficients allows the characterisation of sounds as if they were perceived by the human auditory system. The regularity of the sound is given by the zero crossing rate. Finally, the duration gives an indication of the long or short term emission capacity of a sound source (adult, newborn, device, etc.).

#### 2.3.4. Dimensionality Reduction

A common problem in classification is overfitting. This occurs when a classifier corresponds too closely or exactly to a particular set of data, and may therefore fail to fit additional data [21]. This can be due to an excessively high number of features describing each sample. To prevent this situation and reduce the dimensions *p* of our feature set, we apply principal component analysis (PCA) [22].

Before PCA, a transformation needs to be performed on the feature set in order to work with features on the same scale. This way, the prevalence of a feature in the dimensionality reduction process is avoided. In fact, if the differences between values within a feature are wider than for others, the variance in the whole dataset could be mostly explained by this feature, although that may be incorrect.

To do so, several techniques exist, such as min-max normalisation and standard scaling. In our case, we chose to applied standard scaling, since the maximum and the minimum values that can take each feature may not be present in our dataset. Standard scaling is based on the mean and the variance of each feature. The standardised set of features $Xn_{if}$ is computed for each sample *i* as follows:

(5) $$Xn_{if}=\frac{X_{if}-m_{f}}{s_{f}}$$

where $X_{if}$ is the original value of a feature *f*, with *f*∈ (1,…, *p*). The mean $m_{f}$ and the variance $s_{f}$ of the feature are computed from all samples of the dataset.

Once the PCA was performed on our training database, the principal components representing 95% of the total variance were kept as inputs of classification algorithms.

### 2.4. Cry/Non-Cry Classification

We extracted segments of interest first and summarised them using an ensemble of projected features. From this feature set, six supervised classification approaches were investigated: K-Nearest Neighbours (KNN), Linear Discriminant Analysis (LDA), Logistic Regression (LR), Random Forest (RF), Multi-Layer Perceptron (MLP) and Support Vector Machine (SVM). The main justification to the use of these classical approaches was the amount of annotated data at our disposal (i.e., 637 segments). Indeed, we chose to only learn from real-world data, which led us to consider only classification methods that have proven powers of generalisation, even from size-limited datasets. Secondly, as we cannot formulate any a priori information on the linearity or non-linearity of the most efficient classification, a broad set of classification approaches to identify the best modelling was considered.

Our goal to identify “cry” segments from others, a binary classification was performed. Within this objective, our annotated dataset was composed of 198 cry and 439 non-cry segments. Then, a training/validation set and a testing set were defined, respectively, composed of 60% and 40% of the dataset.

On the training/validation set, a 3-fold cross validation strategy was performed in order to tune the hyper-parameters of the classifiers. The performance of each classifier was evaluated on the testing set through three metrics: precision, recall and accuracy.

Two strategies were considered in order to analyse the impact of the choice of optimising one metric rather than another on classification performance. Hence, models were tuned to reach the highest accuracy (strategy 1) but also to reach the highest precision (strategy 2). In the first case, the idea was to obtain the best balancing between cry and non-cry classification, whereas in the second case, the lowest false positive rate was sought, i.e., recover as few non-cry segments as possible.

## 3. Results

**Figure 2:**
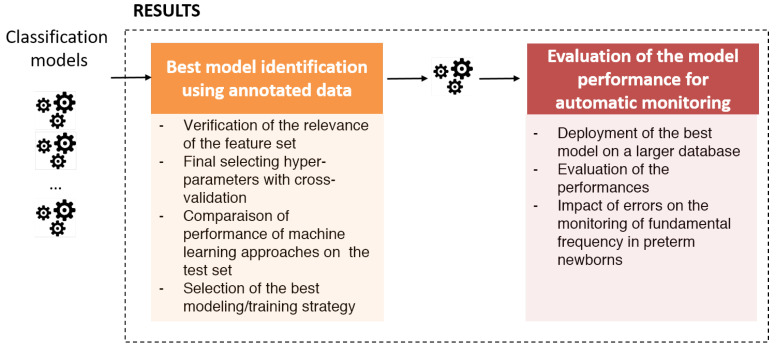
Overview of the evaluation of the approach.

The first part of the results section is dedicated identifying the best classification model to deploy. The second part is focused on the application of this model to the deployment database to evaluate its robustness. Meanwhile, we take the opportunity to study the impact of misclassifications on the estimation of the fundamental frequency. Figure 2 summarises these investigations.

### 3.1. Identification of the Best Classification Model

First, the relevance of the feature set in regard to the classification objective is verified. Then, we present the hyper-parameters of the classification models retained for the two evaluation strategies (described in Section 2.4). Finally, the results in terms of classification performance are presented and the best model is selected.

#### 3.1.1. Relevance of the Feature Set

**Figure 3:**
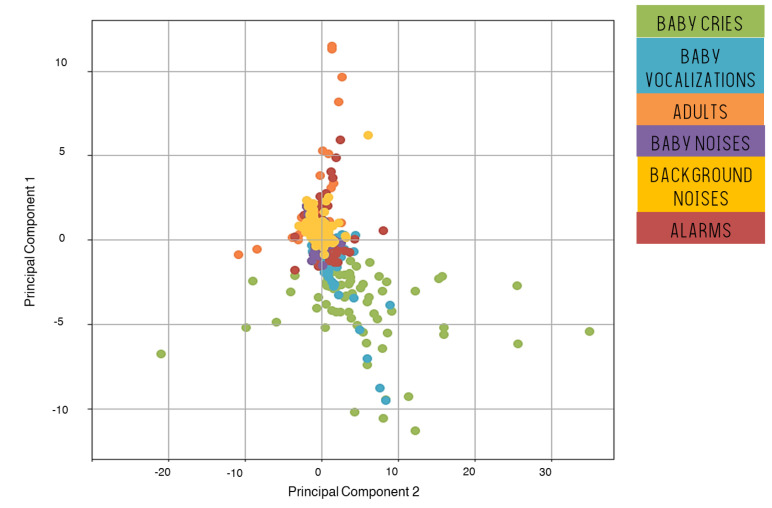
Visualisation of the dataset using the first two principal components.

To give a first insight into the relevance of our approach, features have been projected onto a 2-dimensional space resulting from the PCA analysis (Figure 3).

The two first components contain, respectively, 8.8% and 6.1% of the variance of the dataset, giving a total of 14.9%. Baby cries are mostly located on the bottom of the graph along with some of the baby vocalisations. All other types of sounds tend to be in the upper part. This observation is reassuring regarding our classification purpose, although we may already notice that it will be difficult to discriminate between baby cries and vocalisations on the sole basis of these two dimensions.

**Figure 4:**
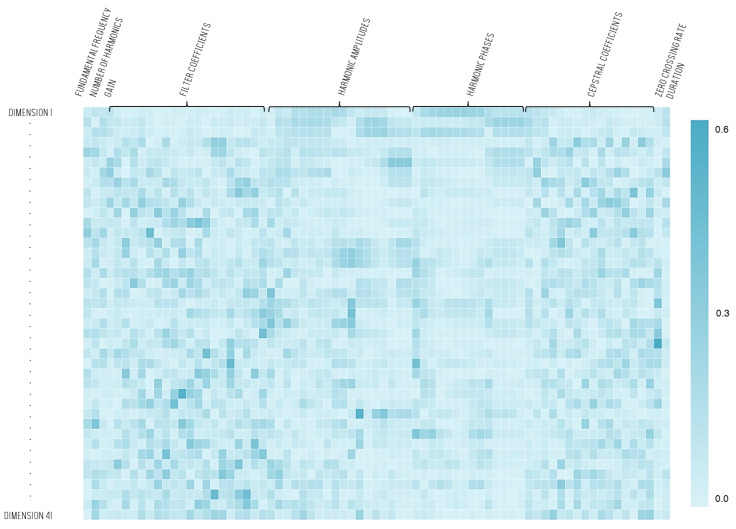
Heatmap reporting the values of the coefficients applied to project the original feature set on 41 principal components.

In a second step, we focused on all the resulting principal components which represent 95% of the total variance or 41 components. The dimensions of the feature set were therefore reduced from 73 to 41. Figure 4 illustrates the coefficients applied to the features in order to perform the projection. A colour represents the value of each coefficient. The deeper the blue, the higher the coefficient applied for a feature.

Globally, each feature contributes to the projection, and none of them stands out from the crowd. One can also see that the most influential features on the first three dimensions are related to the harmonics (the three first lines in Figure 4).

At this stage, we found that the projected feature set contained information from the whole original feature set that can be useful for classification. Indeed, we have seen that the classes seem to be separable in this new projection space. The true suitability of the projected feature set for the extraction of cries will be assessed together with the evaluation of the classifiers in the following sections.

#### 3.1.2. Final Retained Sets of Hyper-Parameters

For each classifier, two sets of parameters resulting in the highest accuracy (strategy 1) and the highest precision (strategy 2) during cross-validation were identified. For that purpose, several parameters and hyper-parameters were tuned using grid search. This time, in order to compare between kernels in SVM, we chose to report the best parameters for the three SVM classifiers: linear, polynomial and Gaussian. A summary of the tests is reported in Table 2.

The parameters are different for different classification strategies. This recalls the fact that this step is inescapable during the learning phase of machine learning and must be investigated to identify the best classifier to use.

#### 3.1.3. Classification Results on the Test Set

**Figure 5:**
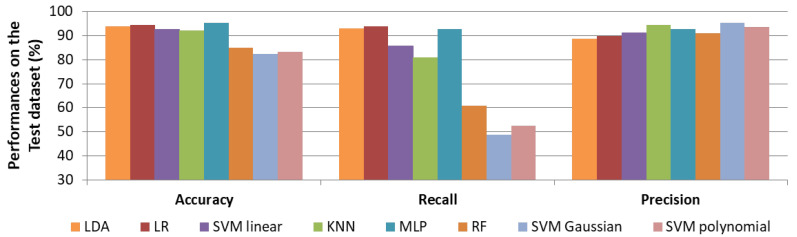
Performances (in %) of cry selection on the test set for each machine learning approach by maximising the accuracy in the learning phase.

In Figure 5, results on the test set regarding strategy 1 (i.e., accuracy) are given. The best results were obtained with MLP, which achieved 95.3% accuracy, 92.9% recall and 92.8% precision. Generally, non-linear algorithms showed weaker results than linear approaches, especially in recall (48.8% for SVM Gaussian, 52.4% for SVM polynomial and 60.7% for RF). This reveals a bad generalisation of those models. The best recall value was obtained by LR with 94.0%, which is slightly higher than the recall of MLP. KNN and SVM linear classifiers performed well, but recall results are lower with values of 85.7% and 81.0%.

**Figure 6:**
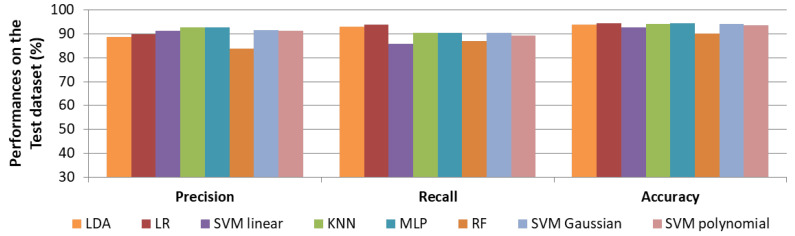
Performances (in %) of cry selection on the test set for each machine learning approach by maximising the precision in the learning phase.

Results for strategy 2 (i.e., precision) are reported in Figure 6. Better generalisation by all models was observed, since values are more stable between metrics. The best precision score was obtained by KNN and reached 92.9%. The highest recall score was once again obtained by LR, 94.1%. Accuracies were high (above 90.2%) for all classifiers. Results with MLP were also really good, since a precision of 92.7%, a recall of 90.48% and an accuracy of 94.5% were reached.

With regard to our objective, a classifier that learned to be precise on cry selection (strategy 2) seems to be the most reasonable choice. Indeed, the goal is to extract cries in order to assess newborn evolution during hospitalisation, in other words, over the long term. In that case, it is much more important to have a high probability that the retained segments are actual cries (even if some are missed) than to have false positives (alarms, adults, etc.). The results of the KNN of the second strategy are in line with this. In fact, it carries high precision while keeping a high recall value. This means that there is also a low chance of missing cries with this model. MLP is also a serious contender, but objectively, with 0.1% more precision, we favour the KNN.

For the remainder of our study, the KNN approach was therefore chosen. A final model was computed by training it to reach the highest possible precision on the whole annotated database.

### 3.2. Evaluation of the Model Performance When Deployed for Monitoring

In this section, the efficiency of the final model in cry extraction on the deployment database is assessed. Secondly, as cries are mainly investigated regarding the fundamental frequency $F_{0}$, the results regarding $F_{0}$ estimations and particularly the impact of misclassifications on these estimations are studied.

#### 3.2.1. Evaluation of the Model Classification Performance during Deployment

**Figure 7:**
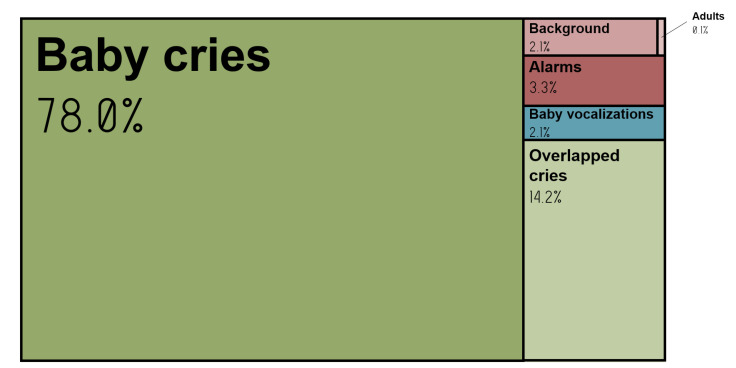
Cry extraction results on the deployment database. Proportions by category of segments automatically labelled as cry by our model.

The whole method was applied on all recordings of the deployment database. After the segmentation step, 495,534 sound events were retained. It was reduced to 5409 segments classified as cry by the KNN model. For practical reasons and to reduce the time needed for annotation, we only analysed the segments that the model extracted as crying. These segments were verified, and percentages of good classifications and misclassifications were computed in regard to two metrics: number of segments and duration of these segments. Results are reported in Figure 7.

Firstly, among the segments, 4221 were revealed to be true positives, giving a precision of 78%. The second most represented segments were ones on which other sound events happened at the same time as crying (14.2% of overlapped cry). If percentages are computed by integrating overlapped cries with cries, precision reaches 92.2% and the error rate drops to 7.8%.

Secondly, alarms represent 3.3% of the misclassified segments, followed by baby vocalisations (2.1%), background noise (2.1%) and adults (0.1%).

To complete these observations, we can also report that processing duration depends on the content of each recording, notably because of the time necessary to perform HNM analysis for classification. It is directly linked to the number of segments issued from the segmentation step and their duration. Here, computational duration ranged from 45 min to 6 h with a mean of 3 h for all 8-hour recordings.

#### 3.2.2. In-Depth Analysis of the Impact of the Crying Extraction Method on the Analysis of Fundamental Frequency

In this part, fundamental frequency estimation is firstly illustrated for cry segments. Then, each type of error is examined to anticipate their impacts on the estimation of the fundamental frequency on a larger scale of analysis (i.e., monitoring).

**Estimations of Fundamental Frequency on True Cry Segments**

To go further, the fundamental frequency estimations obtained on our segments classified as crying were studied. In fact, characterisation of cry through fundamental frequency was shown in the Introduction to be a good indicator of vocal development that can be use to monitor preterm newborn health.

**Figure 8:**
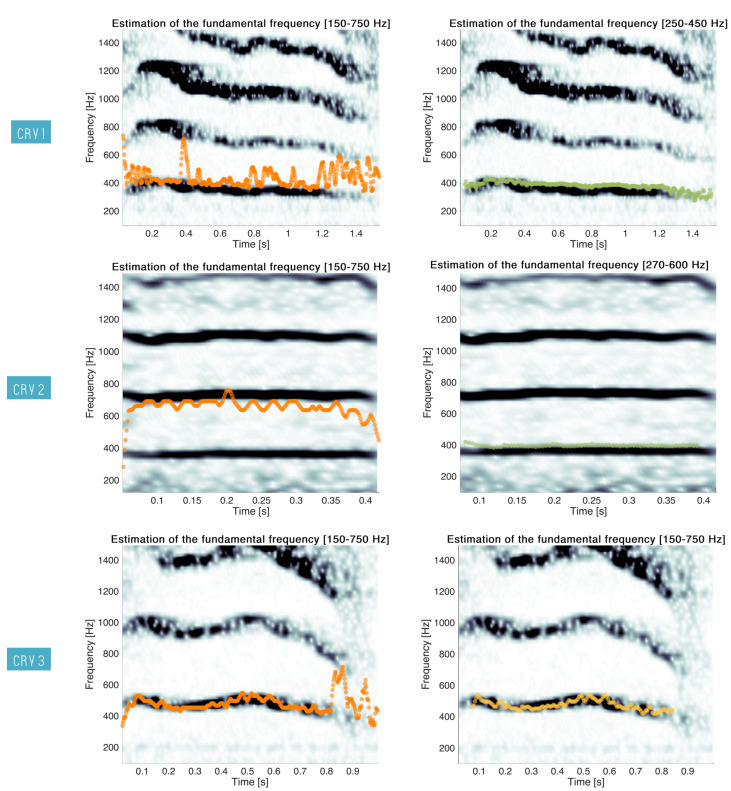
Three examples of cry characterisation using $F_{0}$ estimations. Each time, the estimation either with the fixed band 150–750 Hz (in orange) or with a manually selected band (in green) with smoothing (in yellow) is superimposed on the spectrogram of the cries.

In our method, the fundamental frequency was estimated to be between 150 and 750 Hz in order to be integrated in the feature set and as an input for the HNM analyses. Although we saw that this estimation was relevant for classification, further observations revealed that the band applied for the estimation of $F_{0}$ is not accurate for precise characterisation of a cry. Indeed, the band of analysis had to be adapted for each cry. To illustrate this point, three examples of cries are given in Figure 8.

For Cry 1, several jumps between the fundamental frequency and the first formant can be observed for when the band 150–750 Hz was used. A manual selection of the band 250–450 Hz allowed for a more accurate estimation of $F_{0}$ over the cry. For Cry 2, $F_{0}$ was overestimated and was found on the first formant all along the cry. The use of a cry-specific band (270–600 Hz) provided better estimates.

In Cry 3, noisy parts are at the beginning and at the end of the segment, leading to wrong estimations of $F_{0}$. In [16], the authors proposed to smooth the estimates. This is exemplified with Cry 3 where the smoothed estimation started after and ended before noisy parts of the segment. We can also notice that the smoothing could have been useful to retrieve an accurate estimation of $F_{0}$ with the fixed band in Cry 1.

**Impact of the Sound Superposition on the Estimation of the Fundamental Frequency (Overlapped Cries)**

**Figure 9:**
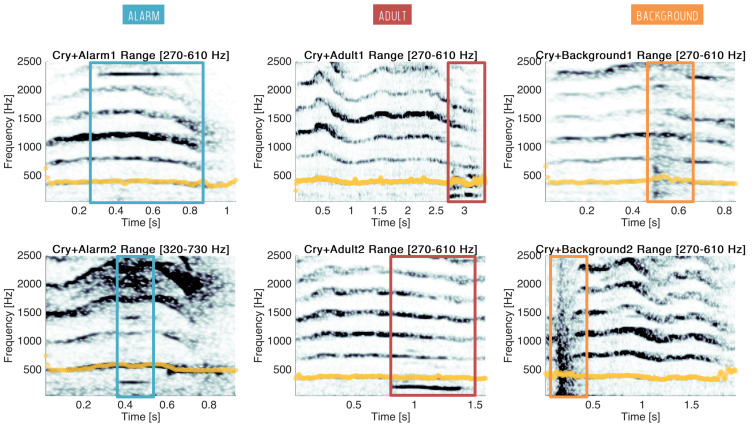
Six examples of cries overlapping with other sounds: two alarms (in blue), two adult voices (in red) and two background noises (in orange). Estimates of the fundamental frequency are superimposed on the spectrogram of the cries (in yellow).

To go one step deeper, the impact of overlapping sounds on the estimation of the fundamental frequency of cries has been studied. Fundamental frequencies were estimated with a manual selection of the estimation band and smoothing for six examples of overlapped cries (see previous section). Results are reported in Figure 9.

First, we can see that an alarm of high (e.g., Cry+Alarm1) or low (e.g., Cry+Alarm2) frequency has no impact on the estimation of the fundamental frequency. Secondly, adults have usually lower fundamental frequencies than babies, from 85 to 180 Hz for men, and from 165 to 255 Hz for women [23]. In most cases, the estimation of $F_{0}$ was well performed (e.g., Cry+Adult2). Nevertheless, the first formant of adult voices can be in the same order as $F_{0}$, and thus may induce errors in the estimation (e.g., Cry+Adult1). Finally, although background noises impact a large frequency band, the estimation of $F_{0}$ remains quite accurate in the affected periods of cries (e.g., Cry+Background1 and Cry+Background2).

**Figure 10:**
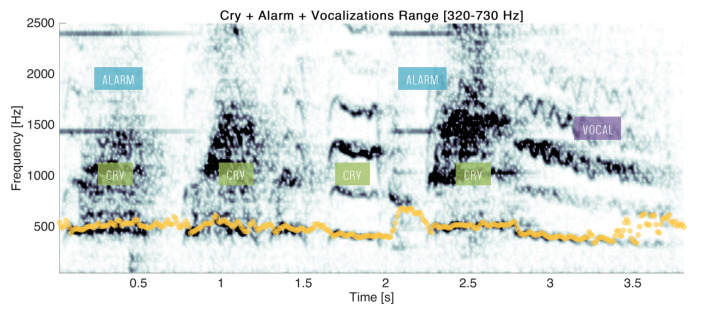
An example of a segment with several sounds and the $F_{0}$ estimates (yellow).

If estimations of $F_{0}$ over a cry are globally not impacted by the overlapping sounds, another limitation coming from the segmentation step can be noticed in cases of multiple events. Indeed, if another sound begins earlier and/or stops after the end of the cry, the algorithm will retrieve all the sound activity as a sole segment, as depicted in Figure 10.

In this example, three types of sounds are present: cry, alarm and vocalisation. First, the segmentation started before and continued after the first cry event because of an alarm. Then, the second cry event began, immediately followed by a new cry and by an alarm. Finally, a fourth cry occurred and was also followed by a vocalisation. In that case, the estimation of the fundamental frequency was performed all along the segment and noisy parts of the spectrum were not avoided or corrected by the smoothing.

**Figure 11:**
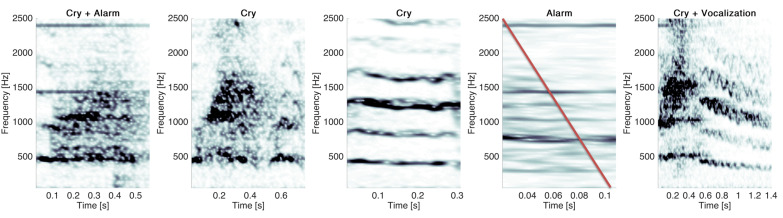
New computation of the cry extraction method (segmentation and classification) for a segment of long duration. The red line indicates the segment that was automatically discarded by the KNN model.

These errors may be identified, since the resulting segments last usually longer (here, more than 3 s). For segments of long duration, it may be relevant to perform the extraction process another time. Results for the segment presented in Figure 10 are reported in Figure 11.

The segmentation step resulted in five segments: one overlapped cry, two cries, one beep and a cry ending with a vocalisation. After classification, the four segments containing cries were retrieved.

**Impacts of Confounding Vocalisations with Cries on the Estimation of the Fundamental Frequency**

**Figure 12:**
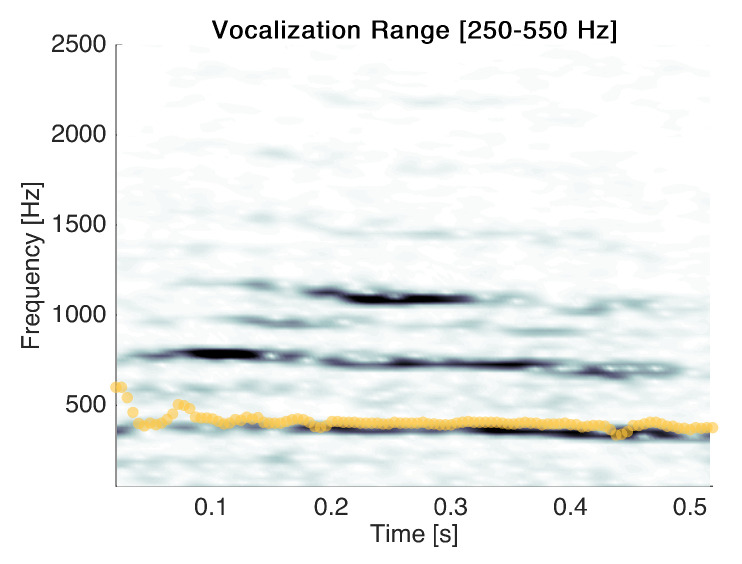
An example of vocalisation and the $F_{0}$ estimates (yellow).

The second type of error made by the classifier was confounding some vocalisations with cry events. An example of misclassified vocalisation is reported in Figure 12.

Misclassified vocalisations have spectra similar to those of cries regarding low frequencies. Among these errors, no oscillatory pattern such as that observed in Figure 10 was observed. In fact, these events are usually short and have values of $F_{0}$ in the same range as cries. Hence, the inclusion of these segments may have no impact on the estimation of $F_{0}$ over long periods of monitoring

**Impact of Other Errors on the Estimation of the Fundamental Frequency**

**Figure 13:**
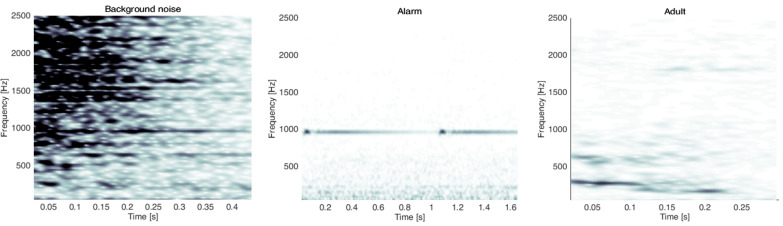
Three examples of misclassified events.

The last errors, representing 5.5%, were between background noises, alarms or adults and cries. An example of a misclassified event from each class is reported in Figure 13.

This time, no estimation of $F_{0}$ was performed since no band of estimation would be relevant. Estimation of the fundamental frequency will be impacted by these kinds of errors if they are too many of them in regard to the number of actual cries.

## 4. Discussion

In this paper, a method based on sound segmentation, feature extraction and machine learning was proposed to automatically extract spontaneous cries of preterm newborns. Methods were set up in order to perform analysis of cry on recording of long duration acquired in real context of NICU. This particular context implies deploying models able to differentiate cry events from non-cry events (alarms, voice of adults).

As a first step, relevant databases for training and evaluating our process had to be collected. Thus, 637 sound segments issued from the sound segmentation of 27 recordings were selected. They were chosen in order to represent the diversity of NICU environments (i.e., type of alarms, different hospitals and types of bed) and the diversity in the preterm newborn population (i.e., sex, GA and PMA). In short, this database was built to retain a performing classification model. A set of features was then defined from each of these segments to be used as inputs of the classification approach. To construct this feature set, we applied a harmonic plus noise model, allowing us to extract features related to the harmonics of the signal while reducing the impacts of noise on them (e.g., MFCCs). We associated with it other classical features, such as zero crossing rate and duration of the segments. Then, we reduced the set of features by means of a PCA. To keep the principal components covering 95% of the variance, 41 components were selected. We saw that each original feature was used to build the projection. Finally, we compared different learning strategies and classification models. On this occasion, we have shown that it is important to tune hyper-parameters of the models by using a metric that is most in line with the objectives of classification. Models trained with a focus on precision had better results. From this analysis, we deduced that the KNN, when trained to reach the highest possible precision, was the best model, with an accuracy of 94%, a precision of 92.9% and a recall of 90.0% on the test set.

In a second step, a larger database (412 h of recording of 23 newborns) was retrieved to ensure the reliability of our final model when deployed. It was found that 92.2% of the segments considered as crying by our model were real crying either alone or with another overlapping sound. Among the false positives, alarms came first (3.3%), followed by vocalisations (2.1%), background noises (2.1%) and adult voices (0.1%). To go further, we studied the potential impact of such errors on the characterisation of crying through the analysis of the fundamental frequency. We have seen that for segments of overlapped cries, the impact on frequency estimation of the interfering sound will be small if a cry-specific selection of the frequency band to perform the analysis is performed. We also found that long duration segments (in which different sound events follow one another) could be resegmented and classified to improve performance. Furthermore, we hypothesised that errors caused by fundamental frequency estimation on neonatal vocalisations could be negligible. Finally, we noted that if the analysis of the $F_{0}$ was done on alarms, background noise or adult voices, the analysis of the vocal development would be biased only if errors are more frequent than cries within the same record.

These results are encouraging and allow us to see new perspectives and avenues for improvement.

Although we have seen that our training database allows us to obtain a well-performing model, increasing the number of examples on which the KNN will rely to make new predictions is a relevant perspective. For that purpose, more segments will have to be annotated. In that sense, the cry extraction process presented here can be used to provide an automatic pre-annotation of segments to facilitate the construction of a large real-world database. In addition, our team has developed an annotation tool that will facilitate this task for future studies (Met-Montot B., Cabon S., Porée F., Carrault G. SoundAnnot: Sound Annotation Tool. IDDN.FR.001.020001.000.S.P.2021.000.31230 (15 July 2020)).

The second area for improvement concerns segmentation. In this study, we applied a method proposed by Orlandi et al. [14] that is not cry-selective, since all types of sound are extracted. During the Digi-NewB project, audio and video data recorded were simultaneously acquired. Hence, we are currently developing a new approach where we combine the motion information with the audio segmentation as the baby moves while crying [24]. This would allow us to reduce classification errors but also to be more robust if several babies are in the same room. Furthermore, we have seen that for segments of long duration, it can be interesting to perform a segmentation a second time. The conditions allowing the automation of this step will have to be studied carefully. Indeed, as the duration of a newborn’s cry is directly linked to its pulmonary and vocal capacities, so it would be unhelpful to lose this piece of information by resegmenting segments containing cries alone.

To finish, the feature extraction step can also be improved. Indeed, as we saw that a cry-specific band associated with smoothing leads to better $F_{0}$ estimates, it will be relevant to feed the HNM with more accurate estimations. We recently proposed a method based on edge detection on spectrogram images to tackle this issue [25]. The other possible improvement is to increase the number of features issued from the HNM analysis, for example, by calculating the derivatives of MFCC (delta and delta–delta) [8] and by performing the analysis in a band of higher frequency (i.e., which would allow one to characterise the high frequency alarms). In addition, other dimensionality reduction techniques could be studied to optimise performances: supervised techniques such as Linear Discriminant Analysis (LDA), step-wise feature elimination and the genetic algorithm; or unsupervised techniques such as t-Stochastic Neighbour Embedding (t-SNE) [26]. Moreover, a large real-word dababase will allow us to dive into newer but data-intensive methods. For example, deep neural networks for cry classification using spectrogram as input should be investigated [27,28,29].

## 5. Conclusions

To conclude this work, a new method for the automatic extraction of cries evaluated under real conditions of NICUs was proposed. This is the first time that the HNM method has been considered for spontaneous cry extraction, but also the first time that performance has been analysed with a strong focus on clinical integration. Our approach performs with 92.2% precision when deployed, and relevant characterisation of crying for monitoring purposes seems achievable with it.

## Figures and Tables

**Table 1:** List of the computed features for classification.

Type of Feature	Estimation Method	Number of Instances
Fundamental frequency	HNM	1
Number of harmonics	HNM	1
Harmonic amplitudes	HNM	18
Harmonic phases	HNM	14
Gain	HNM	1
Filter coefficients	HNM	20
Mel-Frequency Cepstral Coefficients	HNM	16
Zero crossing rate	ZCR	1
Duration	Duration	1

**Table 2:** Parameter testing summary. Final selected parameters for accuracy are underlined, whereas final selected parameters for precision are marked in bold.

Method	Parameters
KNN	Number of neighbors ∈ [1, 3, 5, **11**, 15]
	Distance: Manhattan or **Euclidean**
LDA	Solver ∈ [singular value decomposition,
	**least squares solution**, eigenvalue decomposition]
LR	Cut-off ∈ [**0.1**, 0.2, 0.5, 0.7]
RF	Number of trees ∈ [5, 10, 20, 50, 100, **300**]
	Quality split criterion: gini or **entropy**
MLP	Number of hidden layers ∈ [ **1**, 2, 5]
	Number of perceptrons per layers ∈ [1, 2, 5, 10, **20**, 30]
	Activation function ∈ [identity, logistic sigmoid,
	hyperbolic tan, **rectified linear unit**]
SVM linear	No additional parameter
SVM polynomial	degree ∈ [1, 2, **3**, 4]
SVM gaussian	margin ∈ [0.01, 0.1, **1**, 10, 100, 10${}^{3}$, 10${}^{4}$]
	gamma ∈ [0.0001, 0.001, 0.01, 0.1, 1, **5**, 10, 100]

## Data Availability

In accordance with French policy, the data will be shared in the near future through the health-data-hub (https://www.health-data-hub.fr/, accessed on 21 December 2021), where they will be fully available and respect French regulation.

## References

[1] World Health Organization (2012). Born Too Soon: The Global Action Report on Preterm Birth.

[2] Digi-NewB—GCS HUGO—CHU—Monitoring System. http://www.digi-newb.eu.

[3] Cabon S., Porée F., Simon A., Rosec O., Pladys P., Carrault G. (2019). Video and audio processing in paediatrics: A review. Physiol. Meas..

[4] Manfredi C., Bocchi L., Orlandi S., Spaccaterra L., Donzelli G.P. (2009). High-resolution cry analysis in preterm newborn infants. Med. Eng. Phys..

[5] Shinya Y., Kawai M., Niwa F., Myowa-Yamakoshi M. (2014). Preterm birth is associated with an increased fundamental frequency of spontaneous crying in human infants at term-equivalent age. Biol. Lett..

[6] Orlandi S., Garcia C.A.R., Bandini A., Donzelli G., Manfredi C. (2016). Application of pattern recognition techniques to the classification of full-term and preterm infant cry. J. Voice.

[7] Raboshchuk G., Nadeu C., Jančovič P., Lilja A.P., Köküer M., Mahamud B.M., De Veciana A.R. (2018). A Knowledge-Based Approach to Automatic Detection of Equipment Alarm Sounds in a Neonatal Intensive Care Unit Environment. IEEE J. Transl. Eng. Health Med..

[8] Naithani G., Kivinummi J., Virtanen T., Tammela O., Peltola M.J., Leppänen J.M. (2018). Automatic segmentation of infant cry signals using hidden Markov models. EURASIP J. Audio Speech Music. Process..

[9] Abou-Abbas L., Tadj C., Gargour C., Montazeri L. (2017). Expiratory and inspiratory cries detection using different signals’ decomposition techniques. J. Voice.

[10] Ferretti D., Severini M., Principi E., Cenci A., Squartini S. Infant Cry Detection in Adverse Acoustic Environments by Using Deep Neural Networks. Proceedings of the 2018 26th European Signal Processing Conference (EUSIPCO).

[11] Cabon S., Met-Montot B., Porée F., Rosec O., Simon A., Carrault G. Automatic extraction of spontaneous cries of preterm newborns in neonatal intensive care units. Proceedings of the 2020 28th European Signal Processing Conference (EUSIPCO).

[12] Cabon S., Porée F., Cuffel G., Rosec O., Geslin F., Pladys P., Simon A., Carrault G. (2021). Voxyvi: A system for long-term audio and video acquisitions in neonatal intensive care units. Early Hum. Dev..

[13] Abou-Abbas L., Alaie H.F., Tadj C. (2015). Automatic detection of the expiratory and inspiratory phases in newborn cry signals. Biomed. Signal Process. Control.

[14] Orlandi S., Manfredi C., Bocchi L., Scattoni M. Automatic newborn cry analysis: A non-invasive tool to help autism early diagnosis. Proceedings of the Engineering in Medicine and Biology Society (EMBC), 2012 Annual International Conference of the IEEE.

[15] Otsu N. (1979). A threshold selection method from gray-level histograms. IEEE Trans. Syst. Man Cybern..

[16] Manfredi C., Bandini A., Melino D., Viellevoye R., Kalenga M., Orlandi S. (2018). Automated detection and classification of basic shapes of newborn cry melody. Biomed. Signal Process. Control.

[17] Stylianou Y. (1996). Harmonic Plus Noise Models for Speech, Combined with Statistical Methods, for Speech and Speaker Modification. Ph.D Thesis.

[18] Orlandi S., Guzzetta A., Bandini A., Belmonti V., Barbagallo S.D., Tealdi G., Mazzotti S., Scattoni M.L., Manfredi C. (2015). AVIM—A contactless system for infant data acquisition and analysis: Software architecture and first results. Biomed. Signal Process. Control.

[19] Várallyay G. (2006). Future prospects of the application of the infant cry in the medicine. Period. Polytech. Electr. Eng..

[20] Várallyay G. (2007). The melody of crying. Int. J. Pediatr. Otorhinolaryngol..

[21] Coelho L.P., Richert W. (2015). Building Machine Learning Systems with Python.

[22] Jolliffe I. (2011). Principal Component Analysis.

[23] Baken R.J., Orlikoff R.F. (2000). Clinical Measurement of Speech and Voice.

[24] Cabon S., Porée F., Simon A., Met-Montot B., Pladys P., Rosec O., Nardi N., Carrault G. (2019). Audio-and video-based estimation of the sleep stages of newborns in Neonatal Intensive Care Unit. Biomed. Signal Process. Control.

[25] Met-Montot B., Cabon S., Carrault G., Porée F. Spectrogram-based fundamental frequency tracking of spontaneous cries in preterm newborns. Proceedings of the 2020 28th European Signal Processing Conference (EUSIPCO).

[26] Ayesha S., Hanif M.K., Talib R. (2020). Overview and comparative study of dimensionality reduction techniques for high dimensional data. Inf. Fusion.

[27] Xie J., Long X., Otte R.A., Shan C. (2021). Convolutional neural networks for audio-based continuous infant cry monitoring at home. IEEE Sens. J..

[28] Jindal S., Nathwani K., Abrol V. Classification of Infant Behavioural Traits using Acoustic Cry: An Empirical Study. Proceedings of the 2021 12th International Symposium on Image and Signal Processing and Analysis (ISPA).

[29] Salekin M.S., Zamzmi G., Goldgof D., Kasturi R., Ho T., Sun Y. (2021). Multimodal spatio-temporal deep learning approach for neonatal postoperative pain assessment. Comput. Biol. Med..

